# Oncodrive-CIS: A Method to Reveal Likely Driver Genes Based on the Impact of Their Copy Number Changes on Expression

**DOI:** 10.1371/journal.pone.0055489

**Published:** 2013-02-08

**Authors:** David Tamborero, Nuria Lopez-Bigas, Abel Gonzalez-Perez

**Affiliations:** 1 Research Unit on Biomedical Informatics, Department of Experimental and Health Sciences, University Pompeu Fabra, Barcelona, Spain; 2 Institució Catalana de Recerca i Estudis Avançats (ICREA), Barcelona, Spain; University of Texas MD Anderson Cancer Center, United States of America

## Abstract

A well-established approach for detecting genes involved in tumorigenesis due to copy number alterations (CNAs) is to assess the recurrence of the alteration across multiple samples. Expression data can be used to filter this list of candidates by assessing whether the gene expression significantly differs between tumors depending on the copy number status. A drawback of this approach is that it may fail to detect low-recurrent drivers. Furthermore, this analysis does not provide information about expression changes for each gene as compared to the whole data set and does not take into consideration the expression of normal samples. Here we describe a novel method (Oncodrive-CIS) aimed at ranking genes according to the expression impact caused by the CNAs. The rationale of Oncodrive-CIS is based on the hypothesis that genes involved in cancer due to copy number changes are more biased towards misregulation than are bystanders. Moreover, to gain insight into the expression changes caused by gene dosage, the expression of samples with CNAs is compared to that of tumor samples with diploid genotype and also to that of normal samples. Oncodrive-CIS demonstrated better performance in detecting putative associations between copy-number and expression in simulated data sets as compared to other methods aimed to this purpose, and picked up genes likely to be related with tumorigenesis when applied to real cancer samples. In summary, Oncodrive-CIS provides a statistical framework to evaluate the *in cis* effect of CNAs that may be useful to elucidate the role of these aberrations in driving oncogenesis. An implementation of this method and the corresponding user guide are freely available at http://bg.upf.edu/oncodrivecis.

## Introduction

Interpreting the role of copy number alterations (CNAs) in cancer is challenging because it requires unraveling causative aberrations from passenger ones. A currently well-established approach for identifying genes with alterations involved in the disease is to evaluate whether they are recurrently amplified or deleted across multiple tumor samples, and thereafter to use expression data to further refine the evaluation of the potential drivers: although the expression of key genes may be regulated by other mechanisms, an amplification or deletion that does not modify the expression of the altered gene is unlikely to be tumorigenic [1]. This may be performed by comparing the expression of amplified or deleted tumor samples to their diploid counterparts to check whether they show consistent expression changes [2]. However, this approach has some limitations: first, any method aimed at revealing candidate genes based on the frequency with which the alteration occurs is likely, by definition, to underestimate low-recurrent drivers. Second, this analysis does not include the comparison of the expression data of normal samples that may be available. Third, statistical tests comparing the gene expression of two groups do not provide the best framework to assess the magnitude of the change across the whole altered gene set. Moreover, even small expression changes can reach significance if the sample size is large enough (thus this may overestimate the number of genes to include), and two-groups comparison tests tend to not reach significance when the group of samples with CNAs is small, and this may further impair the detection of less-recurrent drivers.

There are other methods that have been already designed to perform an integrative analysis of gene dosage and expression data. Their performance for detecting concordant gene copy number and expression abnormalities has been evaluated by using simulated data in a recent study [3], which has shown that there is still room for improvement of this type of approaches. Therefore, we present Oncodrive-CIS, a novel method to measure the *in cis* effect of copy number changes that may be useful to identify genes involved in tumorigenesis due to CNAs.

We have evaluated the performance of Oncodrive-CIS in two main ways. First, we have compared its accuracy for detecting putative associations between gene dosage and expression with that obtained by ten methods aimed to integrate both gene expression and dosage data evaluated in [3] by using the same benchmarking procedure. Second, we have assessed the results of applying Oncodrive-CIS to real cancer samples using gliobastoma multiforme (GBM) and ovarian serous carcinoma (OSC) data retrieved from The Cancer Genome Atlas Data Portal.

## Results

### Oncodrive-CIS Overview

The rationale of the method is based on two hypotheses: first, a gene driving oncogenesis through copy number changes is more prone to be biased towards overexpression (or underexpression), compared to bystanders; second, the effect of CNAs is better assessed by observing expression changes not only among tumors but also taking into account normal samples data. Briefly, Oncodrive-CIS consists of the following steps: first, an expression impact score measuring the expression deviation of each sample with CNAs as compared to normal samples (EIS_NORMAL_) and tumor diploid samples (EIS_TUMOR_) were calculated for each gene. Second, a standard score measuring the bias of the EIS_NORMAL_ and the EIS_TUMOR_ of that gene as compared to a null model were obtained by using internal sampling (Z_NORMAL_ and Z_TUMOR_, respectively). Finally, these two scores were combined by the Stouffer’s method to obtain a measurement of the gene expression bias due to CNAs as compared to both normal and tumor diploid samples (Z_COMB_). This combined score was used to rank the genes, and therefore, the higher is the ranking of the gene, the larger the bias towards misregulation caused by the copy number change. Expression-biased gene amplifications should present positive Z_COMB_ values (i.e., towards upregulation), whereas deletions should exhibit negative values. Since the magnitude of expression changes measured in gene deletions was lower than that of multi-copy amplifications, Oncodrive-CIS carried out these analyses separately to obtain a fair estimation of their impact. Thus, two ranked lists were produced, each containing the results for genes having either amplification or deletion events.

Oncodrive-CIS was implemented as a Python script (downloadable from http:/bg.upf.edu/oncodrivecis), which takes both the expression and copy number status values for each gene in each sample as input, together with a sample annotation file stating whether they are tumors or normals (see the user guide for further details). It allows several optional configuration parameters, as for instance the number of sampling procedures performed to calculate Z_NORMAL_ and Z_TUMOR_ in each gene. Since amplifications and deletions are evaluated separately, the Oncodrive-CIS produces two output files (one per each alteration-set analysis). They contain the calculated standard scores (i.e., Z_NORMAL_, Z_TUMOR_ and Z_COMB_) per gene, ordered by their Z_COMB_ values (decreasing order for the amplifications assessment output file, and increasing order for deletions, beginning with the most negative value). If no normal samples are available, only Z_TUMOR_ is calculated.

### Oncodrive-CIS Benchmarking

This was performed by the data simulator used in [3]. Briefly, this tool generates random copy number and expression values for a total of 10,000 genes; 90 of them have copy number variations (of several amplitudes and sizes), but only 54 exhibit a consistent expression change (and therefore they form the true positives set). As in the study by Louhimo et al, we generated raw data for a small (n = 15) and a larger (n = 100) sample tumor sets by using three different patterns of dependence between gene dosage and expression, i.e. a lineal, a stepwise and a sigmoid model. Thereafter, we processed the copy number intensities generated by the simulator by the circular binary segmentation algorithm [4]. Since this method smoothed the transition between the intensity peaks of the nearby amplified (or deleted) genes generated by the simulator, this resulted in stating larger regions of copy number changes. On detail, while the simulator generated 90 gene dosage aberrations, the segmentation algorithm stated a mean of 800 genes with CNAs that were subsequently passed to Oncodrive-CIS.

**Figure 1:**
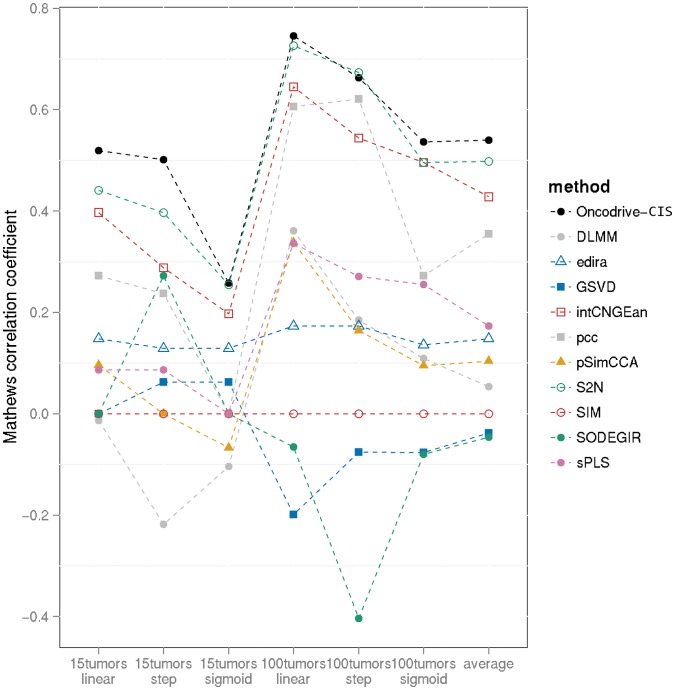
Benchmarking of Oncodrive-CIS using simulated data as compared to other 10 methods aimed to assess the *in cis* effect of copy number changes. Details of the performance of these methods have been retrieved from the supplementary data of the study of Louhimo et al. (see references). The horizontal axis indicates each of the six settings that have been used to generate the simulated data set (the last category depicts the mean of the results obtained in all of them). The vertical axis shows the Matthews correlation coefficient obtained by each method. In the case of Oncodrive-CIS, the performance was checked generating 100 different data sets for each of the simulator settings, thus the mean of the scores obtained in each of them is shown for this method. DLMM: double-layered mixture model; edira: equally directed abnormalities method; GSVD: generalized single value decomposition; intCNGEan: integrative DNA copy number and gene expression analysis; ); pcc: Pearson correlation coefficient; pSimCCA: similarity-constrained probabilistic canonical correlation analysis; S2N: signal-to-noise ratio; SIM: statistical integration of microarrays; SODEGIR: significant overlaps of differentially expressed and genomic imbalanced regions; sPLS: sparse partial least squares regression.

The intersection between the positives identified by Oncodrive-CIS and the 54 true positives generated by the simulator are depicted in Figure S1. To determine them, we selected as Oncodrive-CIS positives those genes that obtained a statistically significant bias towards misregulation, i.e. those genes with a Z_COMB_ equivalent to a corrected p value ≤0.05. Overall, the mean MCC obtained by Oncodrive-CIS across all simulations was 0.54, and it demonstrated better performance in each of the simulation settings when compared to any of the other methods aimed to integrate gene dosage and expression data (Figure 1). Oncodrive-CIS accuracy was worse when applied to the small (n = 15 tumors) data set, although it was indeed satisfactory except for the case in which the dependence between gene expression and copy number followed a sigmoid model. Of note, in the larger data set (n = 100), Oncodrive-CIS obtained a mean sensitivity/specificity of 85/97% when gene expression was lineal to gene dosage, and a mean of 76/97% when a stepwise dependence model was used (Table S1).

### Application to GBM Data

**Figure 2:**
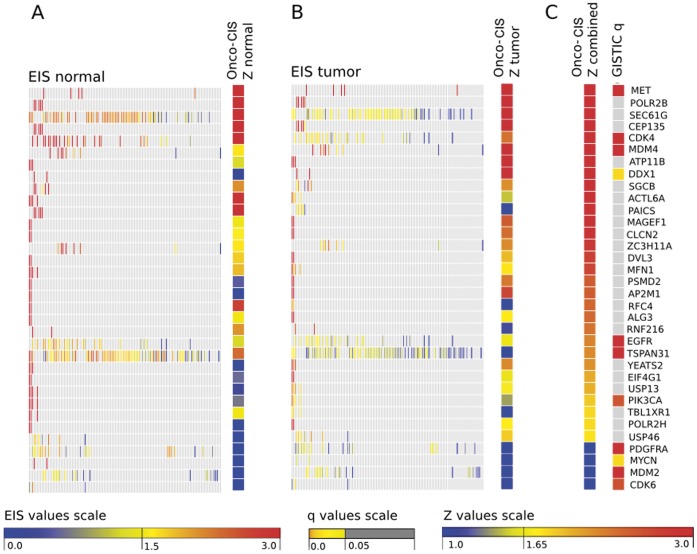
Assessment of the multi-copy amplifications in the gliobastoma multiforme dataset. Results for the top-30 ranking genes according to Oncodrive-CIS analysis are displayed. In addition, we also include 4 genes contained in the Sanger Cancer Gene Census that were significant according to GISTIC method but were not present among the Oncodrive-CIS top-30 ranking. Panel (A): the matrix shows the expression impact scores of the samples with copy number gain compared to normal samples (EIS_normal_); the color bar depicts the standard score measuring the misregulation bias of the samples with copy number gain as compared to normals (Z_normal_). Panel (B) presents the same results for the comparison of samples with gene amplification with diploid tumor samples (EIS_tumor_, Z_tumor_). Note that the depicted Z scores of the present figure are positive in all cases, since a Z value >0 means a shift towards overerexpression. Panel (C): the first color bar depicts the values of the *Z* score used by Oncodrive-CIS to rank the genes, which is obtained by combining the two *Z* scores presented in (A) and (B) using the Stouffer’s method (Z_combined_). This score measures the bias of the samples with copy number alterations towards gene overexpression in comparison to both normal and diploid tumor samples. The second color bar depicts the significance obtained by the GISTIC analysis for that gene.

**Figure 3:**
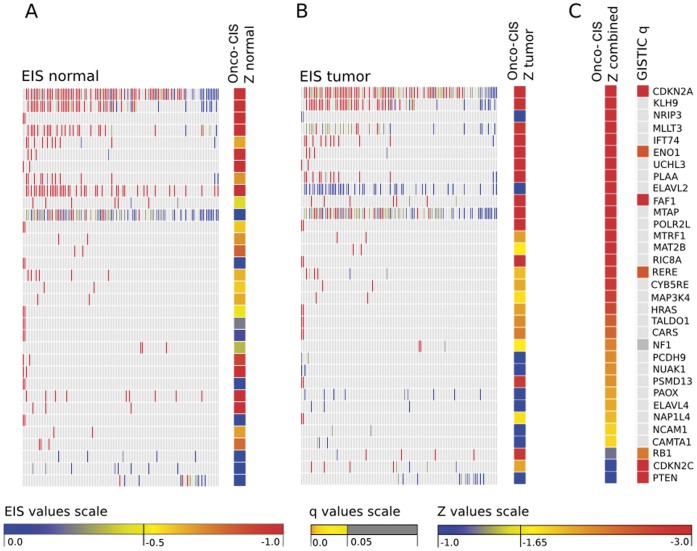
Same as in Figure 1 but for the assessment of homozygous deletions in the gliobastoma multiforme data set. All the *Z* scores obtained for the genes depicted in this figure are negative, i.e. they represent a bias towards underexpression.

**Figure 4:**
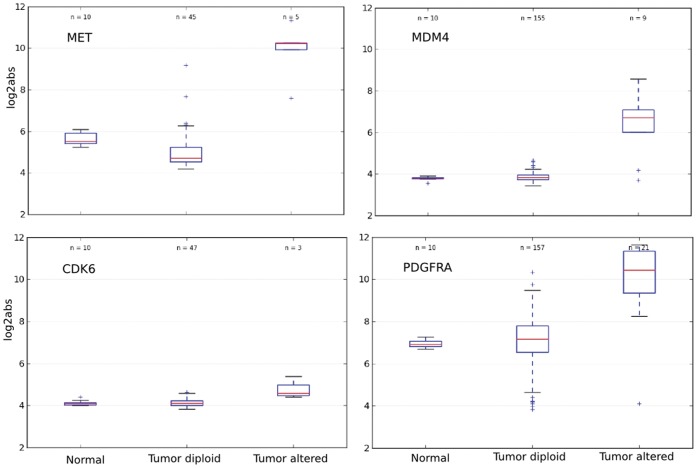
Expression values of several known cancer genes in samples of the gliobastoma data set (log2 transformed absolute expression levels). This figure illustrates the Oncodrive-CIS analysis in different scenarios. MET and MDM4 are severely overexpressed when amplified, compared to both normal and tumor diploid samples, and they appear among the top-30 genes with larger expression change bias according to Oncodrive-CIS. However, the expression change due to CDK6 amplification was moderate and it was classified lower in the ranking list (position 138). PDGFRA was overexpressed when amplified, but the dispersion of the expression values lowered the expression impact scores calculated by Oncodrive-CIS (# 61 on the list). Thus, the two first genes should be considered as positives according to Oncodrive-CIS, whereas the remaining two genes should be considered as negatives according to our analysis criteria.

A total of 206 tumors and 10 normal samples were included in the analysis. Copy number gain or loss in at least two GBM samples occurred in 680 genes. Oncodrive-CIS computed a Z_COMB_ corresponding to a p value ≤0.05 for 77 of these genes (38 due to amplifications and 39 due to deletions). From them, we further examined the top-30 genes that showed larger bias towards overexpression and underexpression, respectively (see Figures 2 and 3). Among them, the following well-known cancer genes were found: MET, CDK4, MDM4, EGFR and PIK3CA (amplifications), and CDKN2A, MLLT3, HRAS, CARS and NF1 (deletions). Other known cancer genes possess lower Z_COMB_ values, either because the expression change observed among samples with CNAs was moderate or because their values were disperse, and thus the expression impact score calculated by Oncodrive-CIS tended to be lower (Figure 4 and Figure S2). Of note, several of these genes were also selected by the GISTIC method [5], which assesses the significance of the recurrence pattern of CNAs across the tumors, stressing the potential benefit of combining Oncodrive-CIS with approaches based on other criteria.

The Oncodrive-CIS ranking noted other potential driver candidates. For instance, the following appeared among the 30 top-ranking genes: DDX1, which is involved in cellular growth and division and previously described as implicated in tumors including neuroblastoma, Wilms tumor, retinoblastoma, testicular carcinoma, and breast cancer [6]; EIF4G1, whose encoded protein enhances the synthesis of mRNA and was described as affecting the tumorigenic potential in a breast cancer cell line [7]; ENO1, which binds to the MYC promoter as a transcriptional repressor, thus likely acting as a tumor suppressor [8]; and MAP3K4, a kinase of CSBP2 and JNK pathways [9]. Further description of the genes of interest from the Oncodrive-CIS top-30 list is included in the Table S2.

Finally, Oncodrive-CIS provided further information about the mechanisms of gene misregulation by observing Z_NORMAL_ and Z_TUMOR_ separately. For instance, ELAVL2, which encodes RNA-binding protein specific to the nervous system, was highly biased towards underexpression among tumor samples regardless of the copy number status. Therefore, downregulation of this gene in GBM would be evoked by other mechanisms besides deletion. Another example is the well-known tumor suppressor CDKN2C, whose expression was low in normal samples but highly increased in tumors with the diploid genotype, probably as a reaction to the aberrant cell state (Figure S3).

### Application to OSC Data

The data set included 480 OSC and 8 normal samples; a total of 9,905 genes presented CNAs in at least two of these tumor samples. Among the 30 genes with the greatest bias in their expression change according to Oncodrive-CIS were CCNE1 and PTEN due to amplifications and CDKN2A, NF1, and NCOA4 due to deletions (Figures S4, S5 and S6). Other potential driver candidates in the Oncodrive-CIS top-30 list were the following: ATAD2, which induces the expression of a subset of estradiol target genes such as CCND1 or MYC, is up-regulated in several other tumor types, and is involved in estrogen-induced cell proliferation and cell-cycle progression of breast cancer cells [10]; BOP1, which is related to the cell cycle and contributes to colorectal tumorigenesis [11]; GLTSCR2, which encodes a PTEN interacting protein and has been associated with low tumor growth and better patient survival in colorectal and esophageal cancers [12]; and MTMR9, which may act to control cell proliferation and has demonstrated prognostic significance for esophageal adenocarcinoma [13]. Further details about the genes of interest on the Oncodrive-CIS top-30 list for OSC are provided in Table S3. Of note, 7 genes that appeared in this OSC list were also present in the top-30 ranking obtained for the GBM data set, including the known cancer genes CDKN2A and NF1 as well as RFC4, a gene encoding a protein associated with BRCA1 that may sensor abnormal DNA structures and/or regulate post-replication repair processes [14].

Observation of Z_NORMAL_ and Z_TUMOR_ pointed out different gene misregulation patterns also in the OSC data set. For instance, the gene encoding the TTK dual specificity protein kinase was upregulated among OSC samples regardless of the CNA status, and CDKN2A expression was low in normal samples but was highly increased in tumors if not deleted (Figure S7).

## Discussion

Oncodrive-CIS may help to elucidate the role of amplified or deleted genes in cancer, since dosage-sensitive genes driving oncogenesis through CNAs should be more biased towards misregulation than genes bearing passenger alterations. For each gene, Oncodrive-CIS measured the magnitude of the expression change caused by copy number changes as compared to a null model obtained by internal sampling. There are several strengths of this method. First, it did not examine the frequency of the alteration across samples and therefore detection of low-recurrent driver alterations was not impaired. Note that the number of samples with CNAs in the Oncodrive-CIS top-ranking genes varies widely for both GBM and OSC data set analysis, and most of those having lower alteration frequency were not selected by GISTIC. Second, amplifications and deletions were evaluated separately to obtain a fair ranking of genes, because the expression change measured in deletions was lower than the one obtained from multi-copy amplifications. Third, the expression of genes in tumor samples was analyzed according to the copy number status but was also compared to normal samples, thus better revealing the gene misregulation role of CNAs in cancer cells. Note that the Z_COMB_ score is calculated by using the same weigh for both Z_NORMAL_ and Z_TUMOR_ values, so the normal data should be reliable in order to avoid unfair calculations. And finally, it should be emphasized that the relationship between expression changes and the actual functional impact is complex: a large expression change in a certain gene might have no significant consequences whatsoever (this would lead to an Oncodrive-CIS false positive) whereas a gene with moderate misregulation could greatly influence processes involved in the disease (and may therefore be a false negative of our method).

The use of simulated data demonstrated that Oncodrive-CIS performs better in the detection of concordant gene expression and copy number abnormalities in different scenarios of sample size and gene abnormalities as compared to the other methods aimed to assess the *in cis* effect of CNAs. Of note, the processing of the raw data may affect the performance of the method. We carried out the benchmarking of Oncodrive-CIS after using the circular binary segmentation that, due to the characteristics of the simulated data, overestimated the number of genes with CNAs. This may impair the subsequent analysis performed by Oncodrive-CIS, especially when the change in expression due to CNAs is smoothed (i.e. the sigmoid model). Whether another method for performing the copy number call could further improve the Oncodrive-CIS results is speculative.

On the other hand, we carried out the benchmarking of Oncodrive-CIS using no directed criteria for selecting the positives of the method, since we considered as Oncodrive-CIS positives those genes with a Z_COMB_ equivalent to a statistically significant *p* value. However, the method gives a statistical framework to rank genes according to their misregulation bias, thus the selection of genes can be customized depending on several criteria, as for instance to prioritize the avoidance of false positives by being more stringent with the Z_COMB_ cutoff. When Oncodrive-CIS is applied to real cancer data, many of the genes exhibiting larger misregulation are either known to be related with tumorigenesis or likely candidates to be involved in the disease. To assess the gene misregulation due to copy number changes may be a valid approach for interpreting the role of these aberrations in cancer, but it must be stressed that Oncodrive-CIS is not exclusive but complementary to methods based on other criteria, such as the recurrence pattern of the CNAs. Comparison between the results of Oncodrive-CIS and GISTIC in the GBM and OSC data sets showed several genes that were highlighted by both methods, whereas other genes were supported by only one of them. In this regard, we can make several observations. Recurrent alterations that cause large expression biases may pick up driver genes. Alterations that occur less frequently but with a large expression impact, as well as those with moderate effect on expression but highly recurrent, may also be driver candidates. Finally, alterations that do not modify expression should be passengers regardless of their prevalence across samples. These results could be further refined by evaluating other alterations causing misregulation, as for instance methylation [15] (Figure S8).

In summary, Oncodrive-CIS exhibited better performance in identifying putative associations between gene copy-number and expression as compared to other methods aimed to assess the *in cis* effect of CNAs. When applied to cancer data, many of the genes identified to have the larger misregulation bias due to copy number changes are likely candidates to be tumorigenic. Moreover, the possibility of integrating data of normal samples in the Oncodrive-CIS analysis may provide further insight into the misregulation mechanisms. Taking into account that CNAs usually affect regions with many genes, preselection of those more likely to be involved in the disease by using the present method should refine downstream analyses aimed to elucidate key biological modules targeted by cancer.

## Methods

### Oncodrive-CIS

Oncodrive-CIS consists of the following steps:

1. An expression impact score (EIS) was obtained for each gene in each tumor sample with copy number change. Let *exp* be the expression value of the gene in the altered sample *j*, *M_R_* the median of the gene expression in the reference group (either tumor samples with no alterations or normal tissue samples, see below), and I*QR_A_* and *IQR_R_* the expression inter-quartile range of the group of tumors with CNAs and the reference group, respectively; the EIS for the *i*th gene and *j*th sample was calculated as:
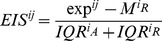

2. The observed EIS of the *i*th gene was then calculated as the median of the EIS^ij^ for all samples *j* (i.e., for all the tumors having CNAs in that gene).
3. Thereafter, a background EIS was calculated for each gene. This was obtained by sampling *n* EIS values from all the EIS^ij^ obtained in step (1), where *n* denoted the number of samples in which the gene *i* had a CNA.
4. Calculation of the background EIS explained above was by default repeated 10,000 times for each gene *i* to estimate an *i*th background EIS model.
5. Finally, the observed *i*th EIS (step 2) was compared with the *i*th background EIS model (step 4) to obtain an *i*th standard score. This *Z* value was used as a measure of the bias of the *i*th gene towards an expression change due to CNAs as compared to the remaining *i* genes:

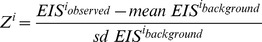
where *sd* means standard deviation.

Two sample groups were used as reference: the group of normal samples and the group of tumor samples in which the gene possessed the diploid genotype. Therefore, two EIS (EIS_NORMAL_ and EIS_TUMOR_) and two *Z* values (ZSupp_NORMAL_ and Z_TUMOR_) were obtained for each gene. EIS_NORMAL_ indicates the expression impact score of a gene computed by comparing the tumor samples with CNAs in that gene to the normal samples used as reference. EIS_TUMOR_ denotes the expression impact score of a gene computed by comparing tumor samples with CNAs to tumor samples without CNAs in that gene. Z_NORMAL_ indicates the bias towards high EIS_NORMAL_, while Z_TUMOR_ indicates the bias towards high EIS_TUMOR_ for a particular gene. Finally, these two *Z* values were combined using the Stouffer method [16] to measure the expression change bias of the samples with CNAs with respect to both normal and tumor diploid samples (Figure S9):
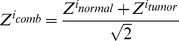



This combined Z score (Z_COMB_) was used by Oncodrive-CIS to rank the genes. Of note, all the samples were included in the analysis regardless whether their expression values were consistent or not with the alteration status (for instance, a sample with a gene deletion which presented higher expression than normal samples). Only those genes with CNAs in at least two tumor samples were by default included in the Oncodrive-CIS analysis.

### Oncodrive-CIS Benchmarking

We have used the data simulator provided in the Supplementary material of the manuscript by Louhimo et al. [3], which generates random copy number and expression raw data for 10,000 genes. By default, 90 of these genes may present copy number variations (of several amplitudes and sizes), but only 54 exhibit a consistent expression change (and therefore they form the true positives set). We processed the copy number intensities generated by the simulator by the binary segmentation method [4], and therefore we used a cutoff of +0.2 and −0.2 for stating a gene gain and loss, respectively [17]. Simulated values were generated 100 times for each of the six data settings used for benchmarking, thus the performance of Oncodrive-CIS is reported as the mean ± standard deviation of the sensitivity, specificity and Matthews correlation coefficient (MCC) [18] obtained in each validation trial. To calculate them, we selected as Oncodrive-CIS positives those genes with a Z_COMB_ equivalent to a corrected p value ≤0.05. Benchmarking results of the other methods already developed to integrate gene expression and dosage data were retrieved from the supplementary data provided by [3].

### Oncodrive-CIS Performance with Two Cancer Data Sets

Copy number status of the GBM and OSC samples were taken from the results of RAE [19], which are available among the data accompanying the MEMo software [20]. Only homozygous deletions or multi-copy amplifications were included; the remaining copy number events were excluded from the analyses. Gene expression values measured by the HT Human Genome U133 were downloaded from the Cancer Genome Atlas Data Portal. To compare the results obtained by Oncodrive-CIS with those obtained by taking into account the recurrence of the CNAs across the tumor samples, we also included the results of the GISTIC analysis [5], which were retrieved from the supplementary data provided by the original GBM and OSC studies [21], [22]. A gene was considered as already known to be cancer-related if it appeared in the Sanger Cancer Gene Census [23]. Heatmaps were constructed using Gitools [24].

## Supporting Information

Figure S1 **The intersection between the positives identified by Oncodrive-CIS and the 54 true positives generated by the simulator.** To carry out the benchmarking, we defined as Oncodrive-CIS positives those genes with a Z_COMB_ equivalent to a p value ≤5%. We simulated 6 different data settings, i.e. sample sizes of 15 or 100 tumors, and a lineal, a stepwise or a sigmoid model of dependence between gene dosage and expression. Since Oncodrive-CIS was benchmarked by using 100 different simulations for each of these 6 settings, the positive gene sets that appeared more frequently in each of them were depicted in the present figure. Venn diagrams have been generated using the BioVenn application (*Hulsen T. et al., BMC Genomics 2008, 9∶488*).
(PDF)

Figure S2 **Expression boxplots for several well-know cancer genes (i.e. included in the Sanger Cancer Gene Census) having copy number alterations in the gliobastoma data set: PIK3CA was overexpressed as compared to both normal and diploid tumor samples when amplified, and MLLT3 is underexpressed as compared to both normal and diploid tumors when deleted.** Both genes obtained a misregulation bias within the top-30 of the Oncodrive-CIS results. On the other hand, amplification of MYCN leads to a small overexpression, whereas samples with amplification of MDM2 presented disperse expression values. Therefore, they obtained a lower ranking in Oncodrive-CIS (positions 84 and 92, respectively). Expression values in all boxplots of the present document are depicted as log2 transformed absolute expression levels.
(PDF)

Figure S3 **Tumor samples with deletion of ELAVL2 were biased towards undexpression as compared to normal samples (Z_NORMAL_ = −7.0) but not as compared to diploid tumors (Z_TUMOR_ >0).** This gene was thus equally misregulated among gliobastoma samples regardless of the gene copy number. On the other hand, expression levels of CDKN2C when deleted were similar regarding to normal samples (Z_NORMAL_ >0) but were biased towards underexpression regarding to diploid tumors (Z_TUMOR_ = −2.1). This could be explained by the fact that this tumor suppressor gene was not significantly acting in normal cells but it reacted to the tumor cell state.
(PDF)

Figure S4 **Same as in** **Figure 1** **(main manuscript) but for the multi-copy amplifications observed in the ovary serous carcinoma data set.**
(PDF)

Figure S5 **Same as in** **Figure 2** **(main manuscript) but for the homozygous deletions observed in the ovary serous carcinoma data set.**
(PDF)

Figure S6 **Expression boxplots for several well-know cancer genes having copy number alterations among the ovary serous carcinoma samples.** Misregulation due to deletions of NF1 and PTEN appeared within the top-30 ranked by Oncodrive-CIS. On the other hand, amplifications in MYC (which occurred in 153 of the tumor samples) showed no substantial effect in the gene expression according to Oncodrive-CIS (this gene appeared in the last part of the ranking list).
(PDF)

Figure S7 **Expression of CDKN2A in normal samples was slight, since it was similar to the expression measured in tumor samples with deletion of the gene (Z_NORMAL_ >0).** However, this tumor suppressor gene was overexpressed in ovarian carcinoma when diploid (Z_TUMOR_ = −13.2). On the other hand, TTK expression was higher among tumors as compared to normal samples, and such overexpression was similar regardless of the gene copy number (Z_NORMAL_ = 13.6, Z_TUMOR_ <0), thus other mechanisms should be acting in such misregulation.
(PDF)

Figure S8 **Expression of CCNE1 in ovarian carcinoma was higher among diploid tumors as compared to normal samples, and it was further overexpressed among tumors with copy gain of the gene (Z_NORMAL_ = 15.7 and Z_TUMOR_ = 4.1).** This could be explained by the presence of additional misregulation mechanisms acting synergistically with copy number alterations.
(PDF)

Figure S9 **Expression boxplots of a dummy gene to illustrate the performance of the Oncodrive-CIS calculations.** The method compares the expression values of tumor samples with CNAs to those of normal samples and also to those of tumors with a diploid genotype for that gene. On detail, Z_NORMAL_ measures the bias towards misregulation in samples with gene copy changes regarding to normal samples, and Z_TUMOR_ measures the bias towards misregulation in samples with gene copy changes regarding to tumors that have two copies of the gene. Z_COMB_ is calculated as a combination of both Z_NORMAL_ and Z_TUMOR_ scores, since those CNAs driving tumorigenesis are expected to shift gene expression both with respect to their normal condition and with respect to the tumor samples in which they appear in double dosage. In this example, a large overexpression is observed among tumor samples with gene amplification as compared to both normal samples and tumor samples in which the gene is diploid. Thus, a large Z_NORMAL_ and Z_TUMOR_ would be obtained, and therefore Z_COMB_ would be consistently large as well.
(PDF)

Table S1 **Mean ± standard deviation obtained by Oncodrive-CIS benchmarking measurements (rows) across the 100 synthetic data sets generated for each of the simulation settings (columns).** MCC means Matthew’s correlation coefficient.
(PDF)

Table S2 **Summary of the genes manually selected as being of interest within the top-30 ranking list obtained by Oncodrive-CIS for the gliobastoma multiforme data set analysis.** Summary extracted from *Omim*, *Entrez Gene*, *UniProtKB/Swiss-Prot* public resources. An * means that the gene is included in the Sanger Cancer Gene Census.
(PDF)

Table S3 **Same as Table S2 but for the Oncodrive-CIS results obtained in the ovarian serous carcinoma data set.**
(PDF)
