# Supplementary material for: Oncodrive-CIS: A Method to Reveal Likely Driver Genes Based on the Impact of Their Copy Number Changes on Expression
Source: PLoS One. 2013 Feb 8;8(2):e55489. doi: 10.1371/journal.pone.0055489 (PMC3568145; doi:10.1371/journal.pone.0055489)
Supplement: Figure S1 — The intersection between the positives identified by Oncodrive-CIS and the 54 true positives generated by the simulator. To carry out the benchmarking, we defined as Oncodrive-CIS positives those genes with a ZCOMB equivalent to a p value ≤5%. We simulated 6 different data settings, i.e. sample sizes of 15 or 100 tumors, and a lineal, a stepwise or a sigmoid model of dependence between gene dosage and expression. Since Oncodrive-CIS was benchmarked by using 100 different simulations for each of these 6 settings, the positive gene sets that appeared more frequently in each of them were depicted in the present figure. Venn diagrams have been generated using the BioVenn application (Hulsen T. et al., BMC Genomics 2008, 9∶488). (PDF) [file pone.0055489.s001.pdf]

Figure S1

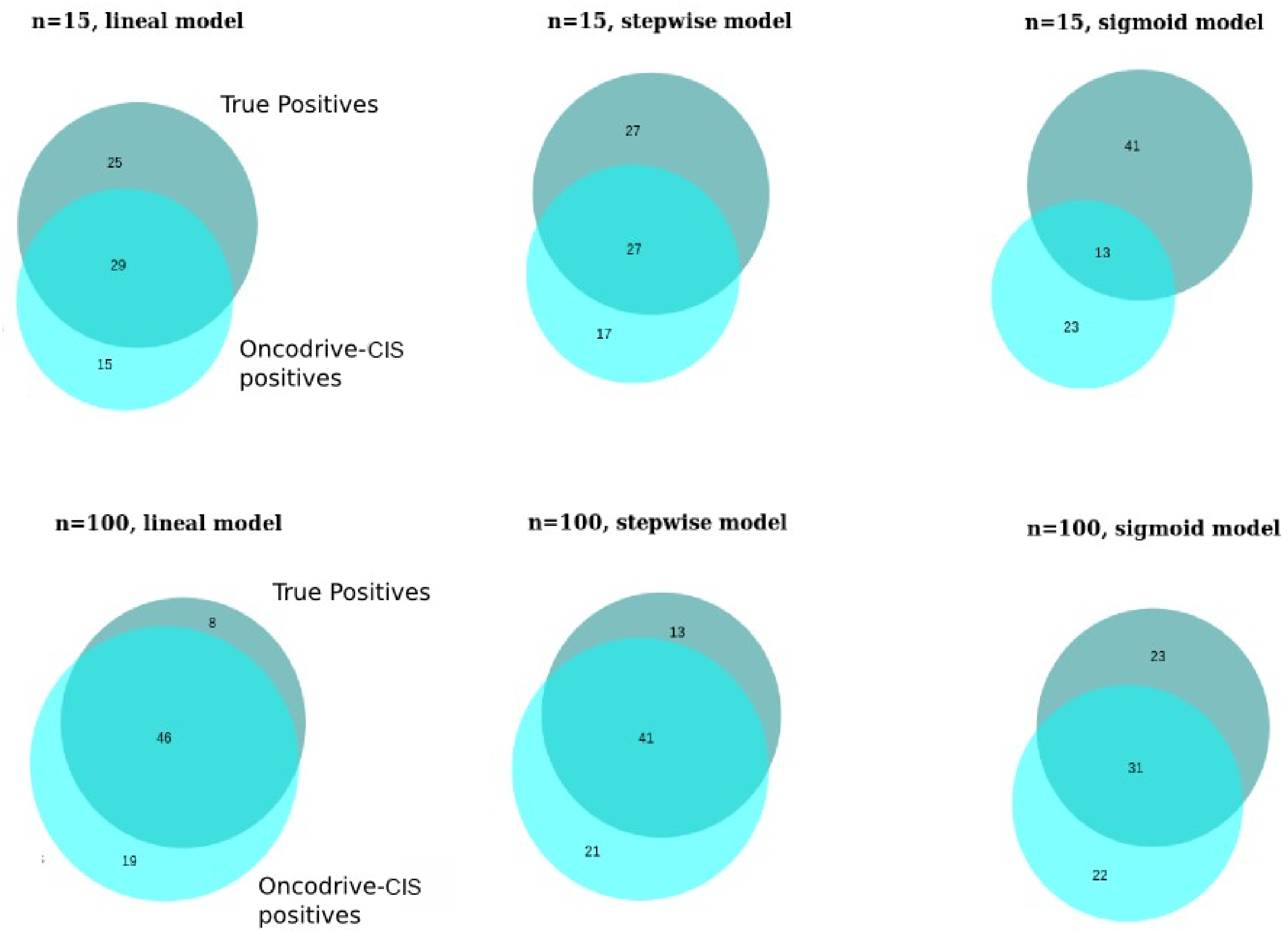
n=15, lineal model
True Positives
25
29
15
Oncodrive-CIS positives
n=15, stepwise model
27
27
17
n=15, sigmoid model
41
13
23
n=100, lineal model
True Positives
8
46
19
Oncodrive-CIS positives
n=100, stepwise model
13
41
21
n=100, sigmoid model
23
31
22
