# Supplementary figures and images for: Oncodrive-CIS: A Method to Reveal Likely Driver Genes Based on the Impact of Their Copy Number Changes on Expression
Source: PLoS One. 2013 Feb 8;8(2):e55489. doi: 10.1371/journal.pone.0055489 (PMC3568145; doi:10.1371/journal.pone.0055489)

**Figure S2**

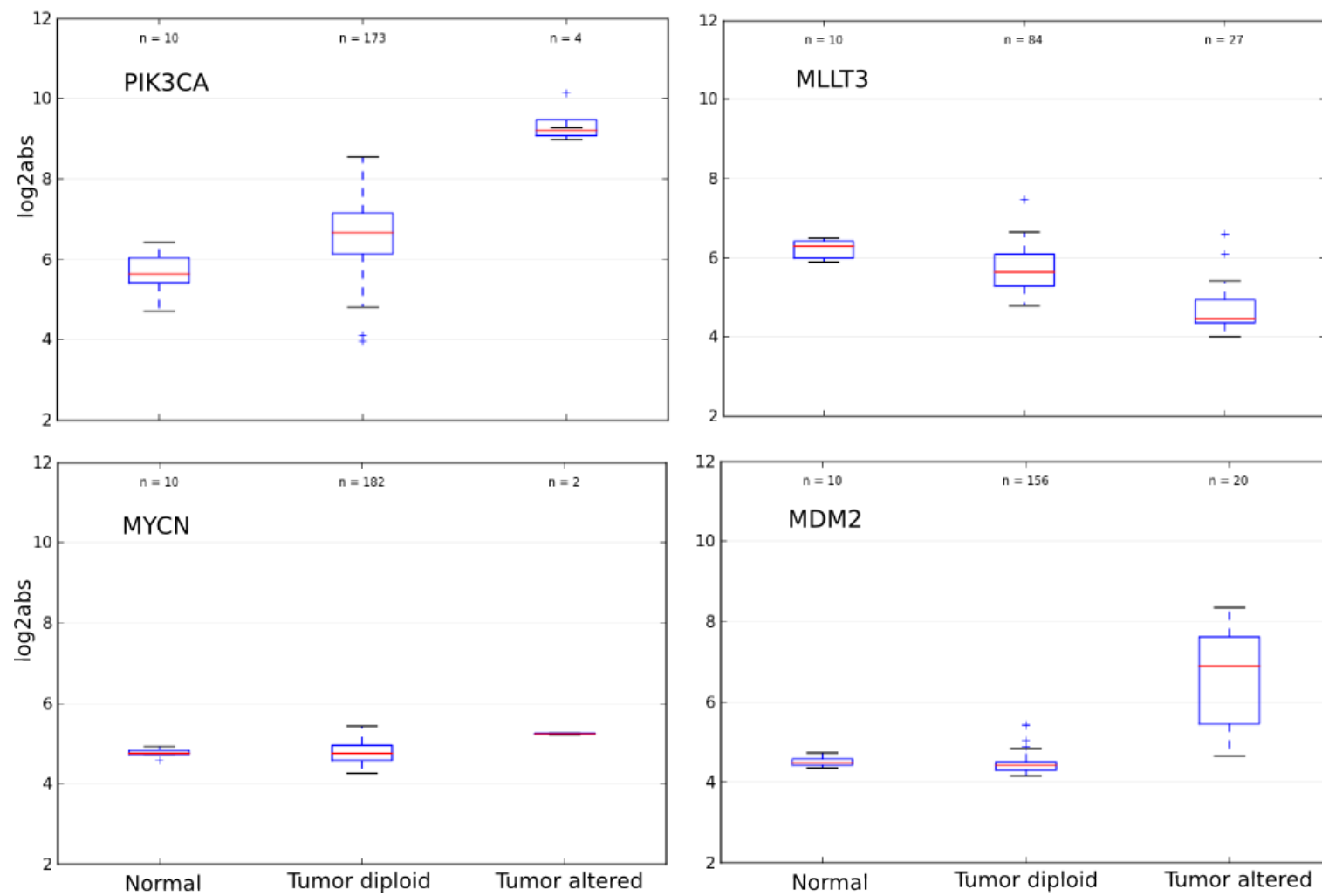

Supplement: Figure S2 — Expression boxplots for several well-know cancer genes (i.e. included in the Sanger Cancer Gene Census) having copy number alterations in the gliobastoma data set: PIK3CA was overexpressed as compared to both normal and diploid tumor samples when amplified, and MLLT3 is underexpressed as compared to both normal and diploid tumors when deleted. Both genes obtained a misregulation bias within the top-30 of the Oncodrive-CIS results. On the other hand, amplification of MYCN leads to a small overexpression, whereas samples with amplification of MDM2 presented disperse expression values. Therefore, they obtained a lower ranking in Oncodrive-CIS (positions 84 and 92, respectively). Expression values in all boxplots of the present document are depicted as log2 transformed absolute expression levels. (PDF) [file pone.0055489.s002.pdf]

**Figure S3**

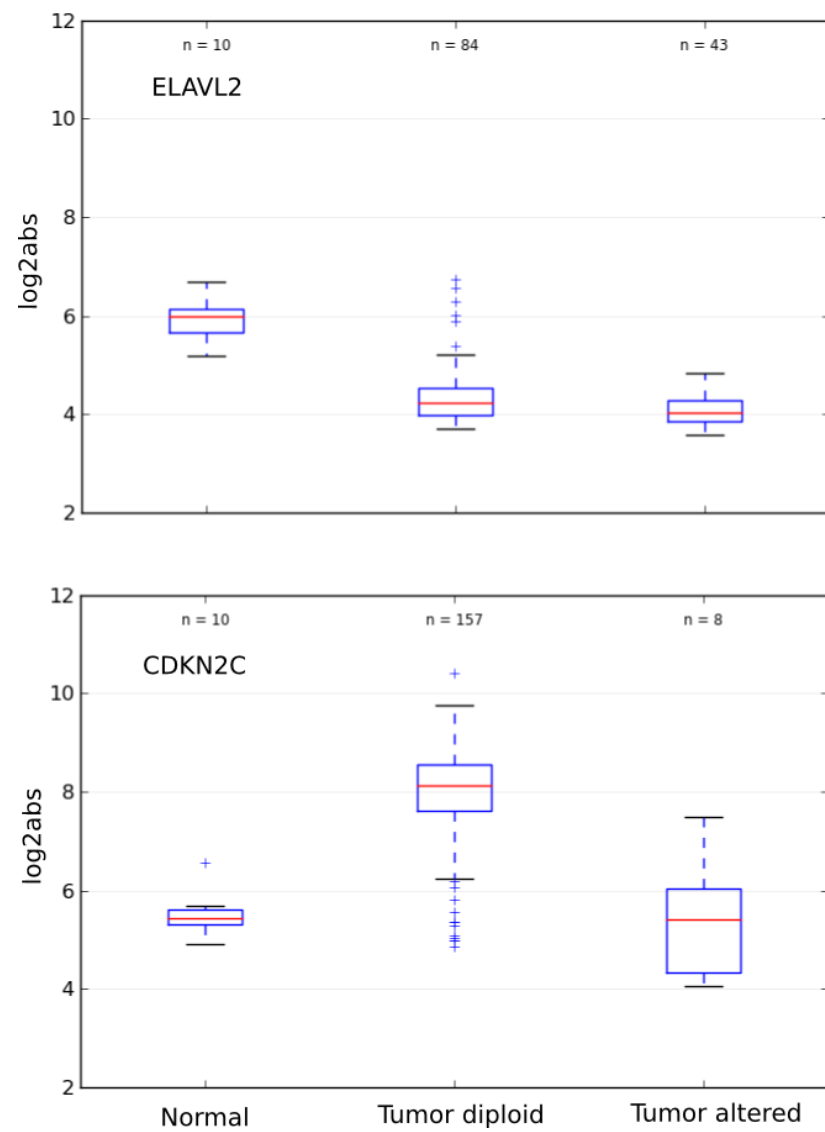

Supplement: Figure S3 — Tumor samples with deletion of ELAVL2 were biased towards undexpression as compared to normal samples (ZNORMAL = −7.0) but not as compared to diploid tumors (ZTUMOR >0). This gene was thus equally misregulated among gliobastoma samples regardless of the gene copy number. On the other hand, expression levels of CDKN2C when deleted were similar regarding to normal samples (ZNORMAL >0) but were biased towards underexpression regarding to diploid tumors (ZTUMOR = −2.1). This could be explained by the fact that this tumor suppressor gene was not significantly acting in normal cells but it reacted to the tumor cell state. (PDF) [file pone.0055489.s003.pdf]

**Figure S4.**

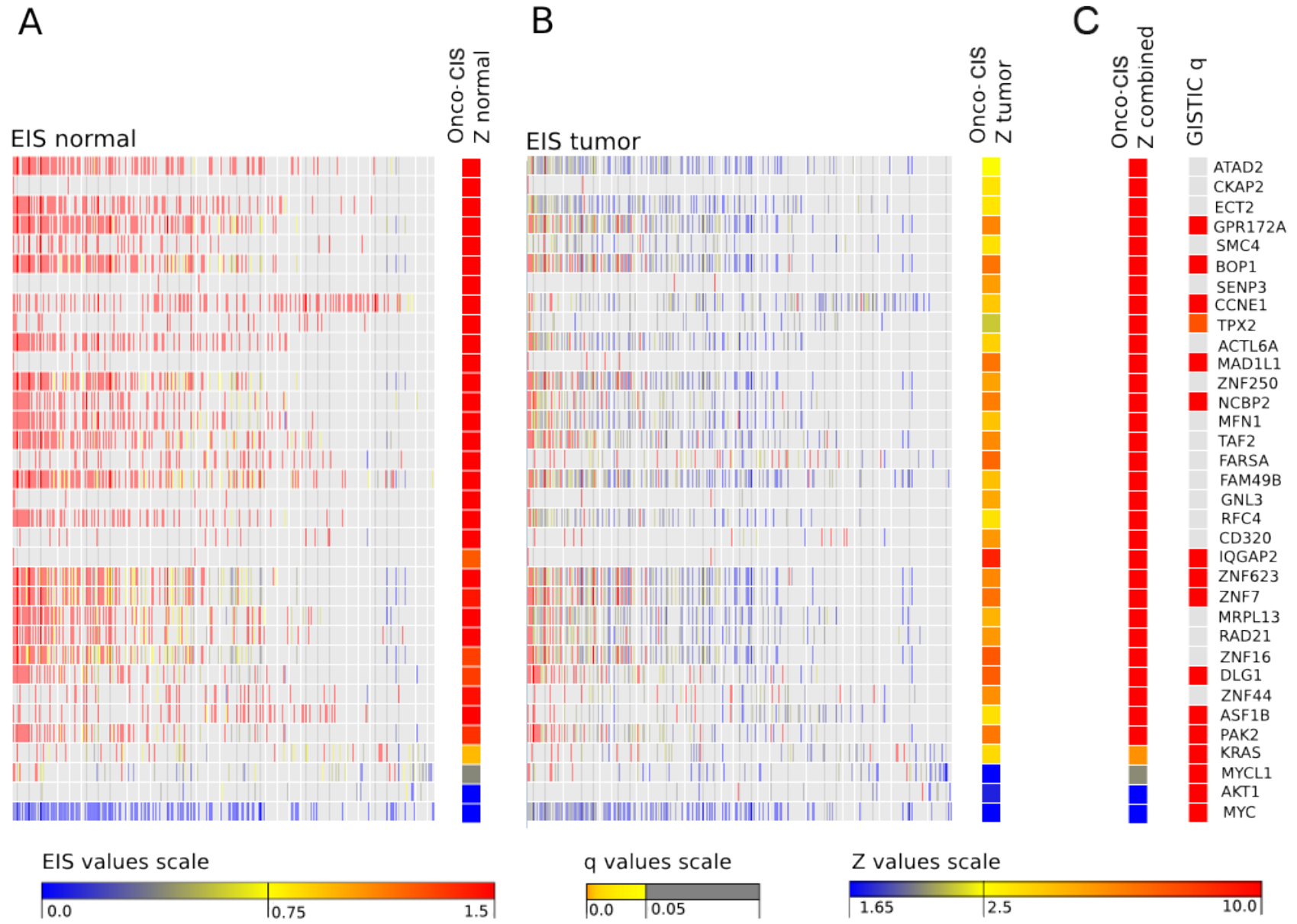

Supplement: Figure S4 — Same as in Figure 1 (main manuscript) but for the multi-copy amplifications observed in the ovary serous carcinoma data set. (PDF) [file pone.0055489.s004.pdf]

Figure S5

A

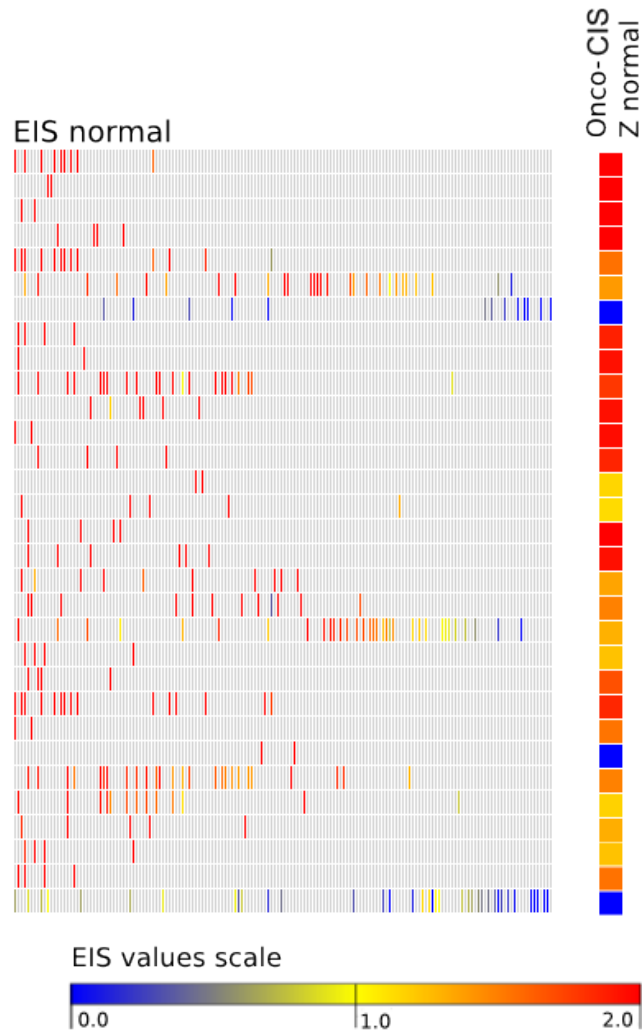

B

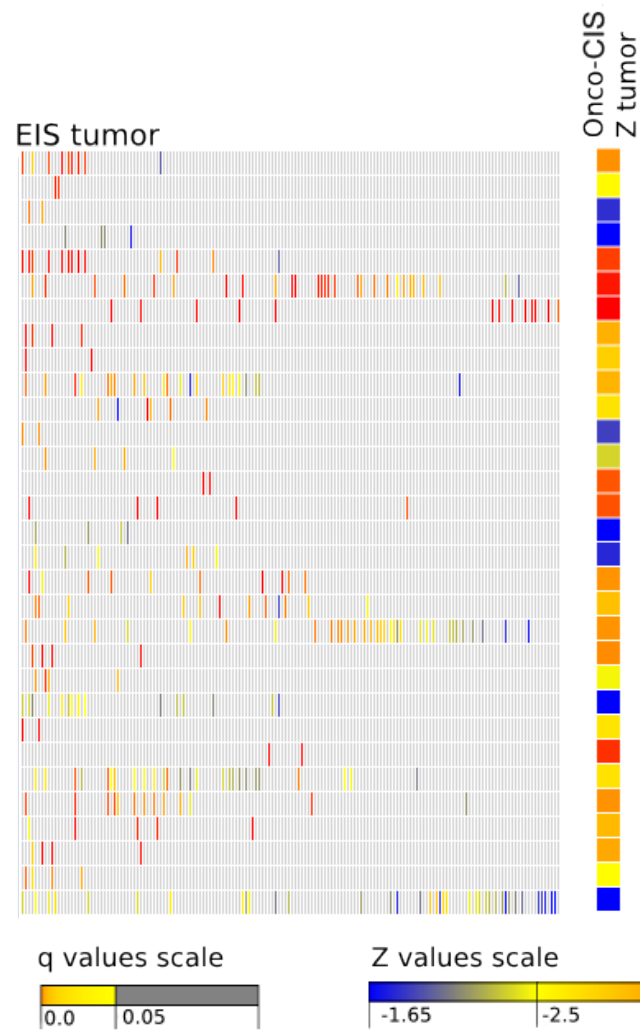

C

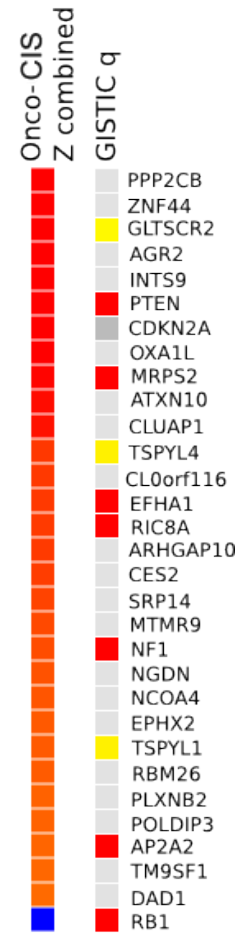

Supplement: Figure S5 — Same as in Figure 2 (main manuscript) but for the homozygous deletions observed in the ovary serous carcinoma data set. (PDF) [file pone.0055489.s005.pdf]

**Figure S6.**

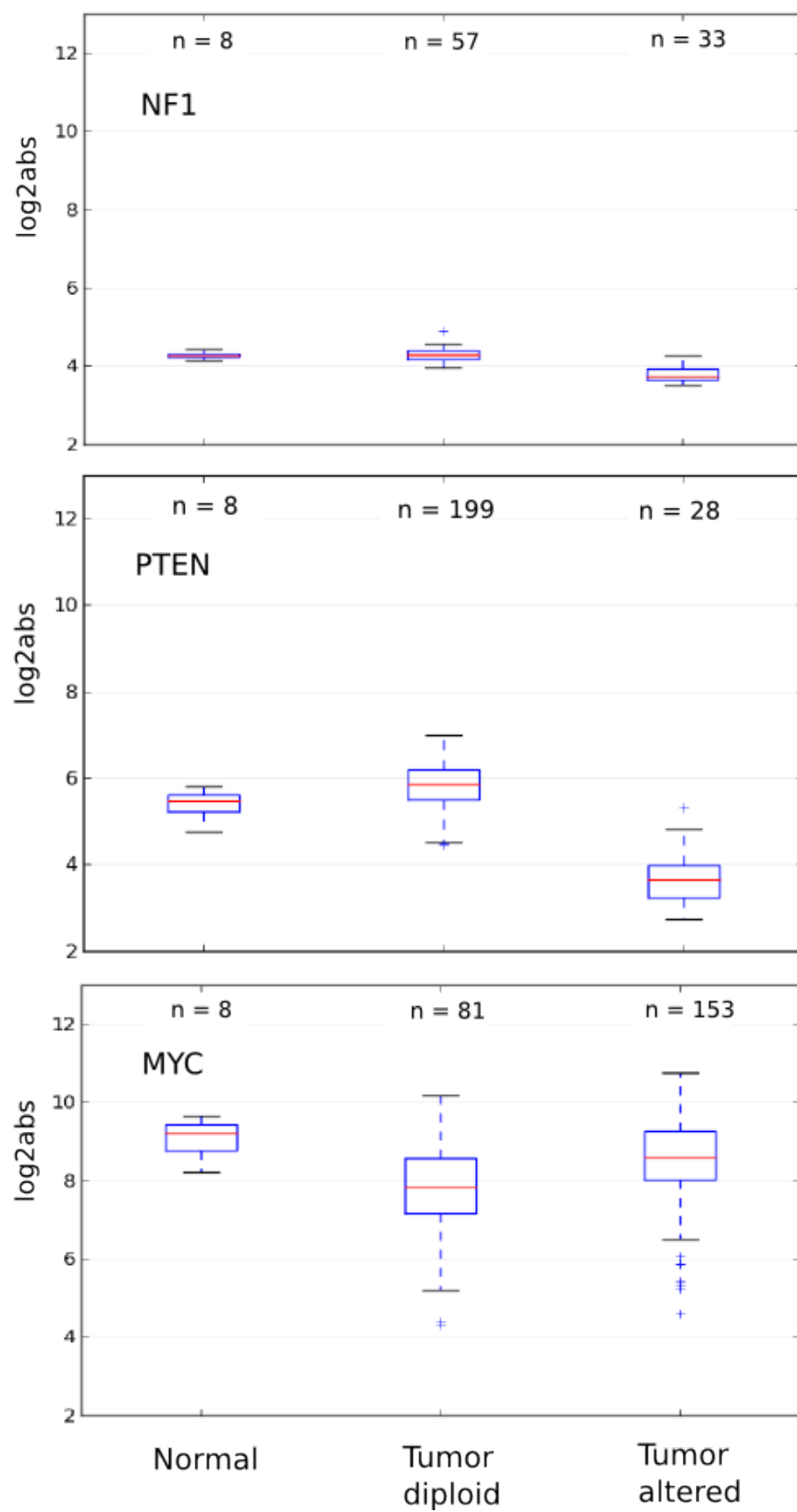

Supplement: Figure S6 — Expression boxplots for several well-know cancer genes having copy number alterations among the ovary serous carcinoma samples. Misregulation due to deletions of NF1 and PTEN appeared within the top-30 ranked by Oncodrive-CIS. On the other hand, amplifications in MYC (which occurred in 153 of the tumor samples) showed no substantial effect in the gene expression according to Oncodrive-CIS (this gene appeared in the last part of the ranking list). (PDF) [file pone.0055489.s006.pdf]

**Figure S7.**

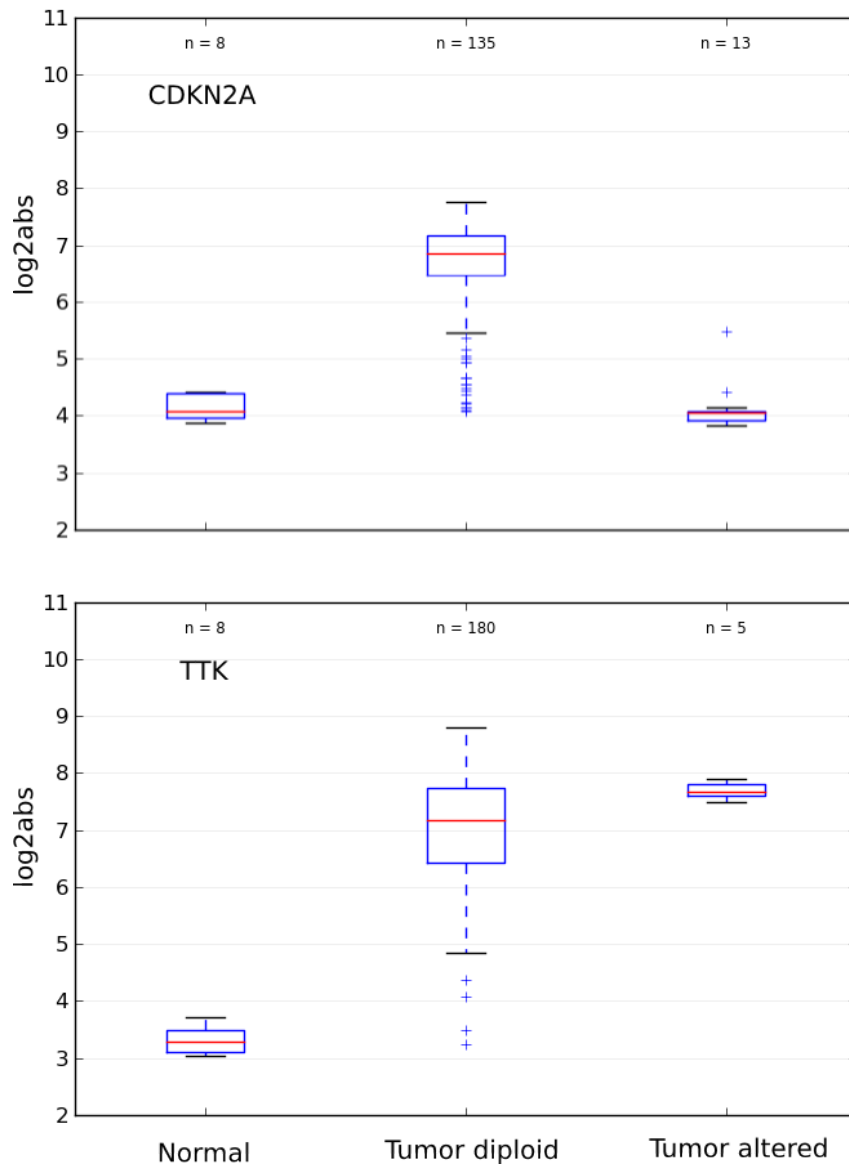

Supplement: Figure S7 — Expression of CDKN2A in normal samples was slight, since it was similar to the expression measured in tumor samples with deletion of the gene (ZNORMAL >0). However, this tumor suppressor gene was overexpressed in ovarian carcinoma when diploid (ZTUMOR = −13.2). On the other hand, TTK expression was higher among tumors as compared to normal samples, and such overexpression was similar regardless of the gene copy number (ZNORMAL = 13.6, ZTUMOR <0), thus other mechanisms should be acting in such misregulation. (PDF) [file pone.0055489.s007.pdf]

**Figure S8.**

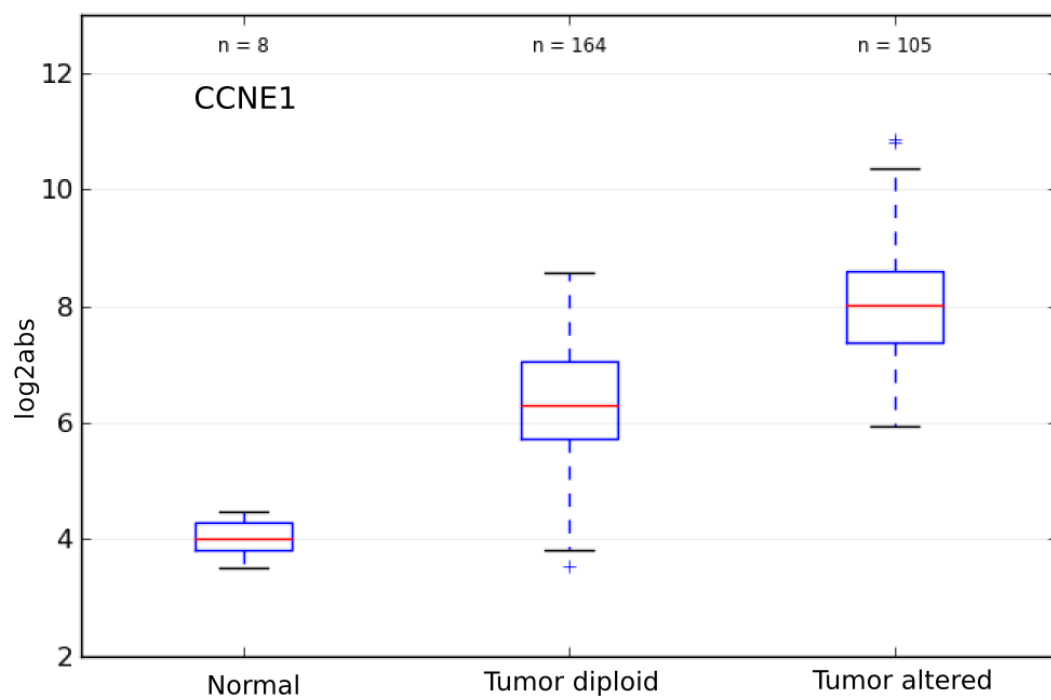

Supplement: Figure S8 — Expression of CCNE1 in ovarian carcinoma was higher among diploid tumors as compared to normal samples, and it was further overexpressed among tumors with copy gain of the gene (ZNORMAL = 15.7 and ZTUMOR = 4.1). This could be explained by the presence of additional misregulation mechanisms acting synergistically with copy number alterations. (PDF) [file pone.0055489.s008.pdf]

**Figure S9.**

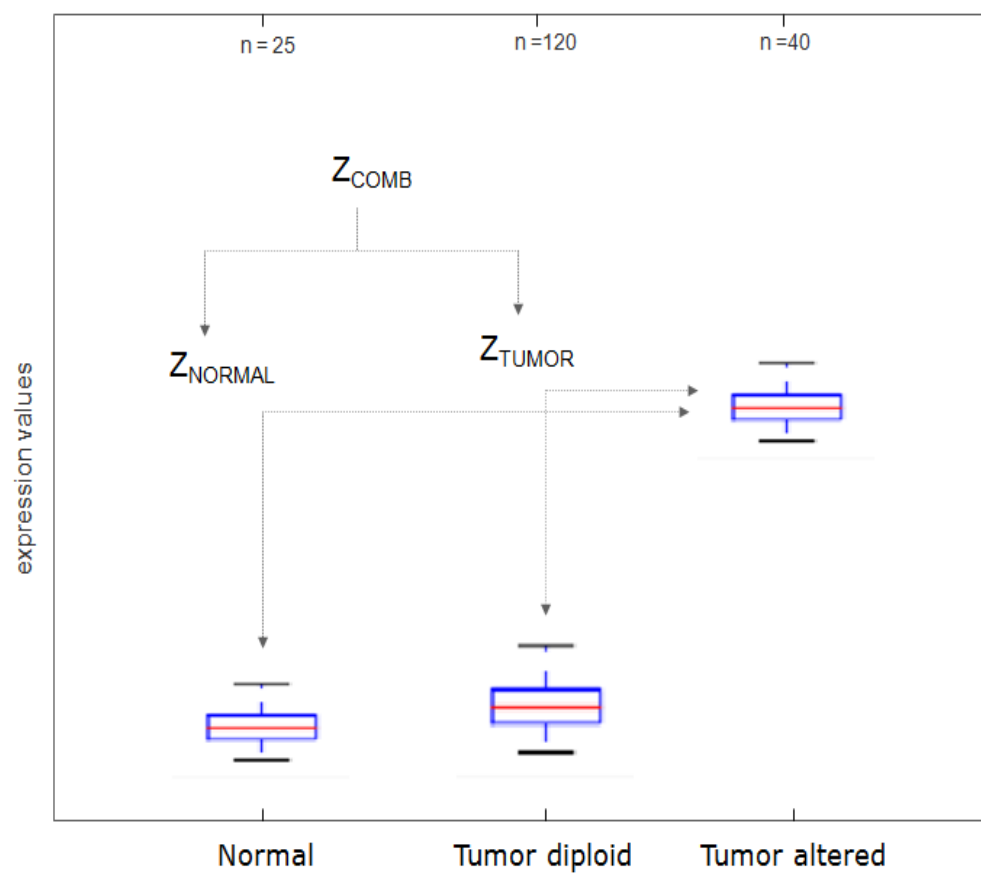

Supplement: Figure S9 — Expression boxplots of a dummy gene to illustrate the performance of the Oncodrive-CIS calculations. The method compares the expression values of tumor samples with CNAs to those of normal samples and also to those of tumors with a diploid genotype for that gene. On detail, ZNORMAL measures the bias towards misregulation in samples with gene copy changes regarding to normal samples, and ZTUMOR measures the bias towards misregulation in samples with gene copy changes regarding to tumors that have two copies of the gene. ZCOMB is calculated as a combination of both ZNORMAL and ZTUMOR scores, since those CNAs driving tumorigenesis are expected to shift gene expression both with respect to their normal condition and with respect to the tumor samples in which they appear in double dosage. In this example, a large overexpression is observed among tumor samples with gene amplification as compared to both normal samples and tumor samples in which the gene is diploid. Thus, a large ZNORMAL and ZTUMOR would be obtained, and therefore ZCOMB would be consistently large as well. (PDF) [file pone.0055489.s009.pdf]
