# Supplementary material for: Oncodrive-CIS: A Method to Reveal Likely Driver Genes Based on the Impact of Their Copy Number Changes on Expression
Source: PLoS One. 2013 Feb 8;8(2):e55489. doi: 10.1371/journal.pone.0055489 (PMC3568145; doi:10.1371/journal.pone.0055489)
Supplement: Table S1 — Mean ± standard deviation obtained by Oncodrive-CIS benchmarking measurements (rows) across the 100 synthetic data sets generated for each of the simulation settings (columns). MCC means Matthew’s correlation coefficient. (PDF) [file pone.0055489.s010.pdf]

Table S1.

Mean ± standard deviation obtained by Oncodrive-CIS benchmarking measurements (rows) across the 100 synthetic data sets generated for each of the simulation settings (columns). MCC means Matthew's correlation coefficient.

	n = 15<br>lineal	n = 15<br>stepwise	n = 15<br>sigmoid	n = 100<br>lineal	n = 100<br>stepwise	n = 100<br>sigmoid
Sensitivity	0.520 ±0.021	0.490 ±0.010	0.249 ± 0.008	0.851 ± 0.011	0.758 ±0.004	0.569 ±0.014
Specificity	0.975 ±0.007	0.974 ±0.001	0.969 ±0.001	0.972 ±0.001	0.967 ±0.002	0.969 ±0.001
MCC	0.513 ± 0.018	0.501 ± 0.013	0.258 ±0.011	0.745 ±0.010	0.663 ±0.009	0.536 ±0.017
