# Supplementary material for: Oncodrive-CIS: A Method to Reveal Likely Driver Genes Based on the Impact of Their Copy Number Changes on Expression
Source: PLoS One. 2013 Feb 8;8(2):e55489. doi: 10.1371/journal.pone.0055489 (PMC3568145; doi:10.1371/journal.pone.0055489)
Supplement: Table S2 — Summary of the genes manually selected as being of interest within the top-30 ranking list obtained by Oncodrive-CIS for the gliobastoma multiforme data set analysis. Summary extracted from Omim, Entrez Gene, UniProtKB/Swiss-Prot public resources. An * means that the gene is included in the Sanger Cancer Gene Census. (PDF) [file pone.0055489.s011.pdf]

Table S2. Summary of the genes manually selected as being of interest within the top-30 ranking list obtained by Oncodrive-CIS for the glioblastoma multiforme data set analysis. Summary extracted from *Omim*, *Entrez Gene*, *UniProtKB/Swiss-Prot* public resources. An * means that the gene is included in the Sanger Cancer Gene Census.

	**AMPLIFICATIONS**
**Gene**	**Description**
MET*	The proto-oncogene MET product is the hepatocyte growth factor receptor and encodes tyrosine-kinase activity.
CDK4*	Cyclin-dependent kinases are heteromeric serine/threonine kinases that control progression through the cell cycle in concert with their regulatory subunits, the cyclins.
MDM4*	Inhibits p53/TP53- and TP73/p73-mediated cell cycle arrest and apoptosis by binding its transcriptional activation domain. Inhibits degradation of MDM2. Can reverse MDM2-targeted degradation of TP53 while maintaining suppression of TP53 transactivation and apoptotic functions.
DDX1	A member of the DEAD box protein family which is involved in cellular growth and division. Tanaka et al [2009] concluded that DDX1 is a stem cell regulator that contributes to testicular germ cell tumors when overexpressed.
MAGEF1	Member of the MAGE superfamily of tumor antigens, this gene is expressed in many tumor types including ovarian, breast, cervical, melanoma and leukemia [Brad Stone et al 2001]
DVL3	Homologous of the Drosophila dishevelled gene regulates cell proliferation, acting as a transducer molecule for developmental processes, including segmentation and neuroblast specification. Upstream regulator of the Wnt pathway.<br>Enhancement of DVL-3 mediated Wnt signalling pathway by Rac1b overexpression leads to colorectal tumor progression [Esufali et al 2008].<br>Misregultation of Dishevelled 3 (Dvl3) enhances cell migration and invasion in hepatocellular carcinoma [http://hub.hku.hk/handle/10722/136002]
PSMD2	In addition to participation in proteasome function, this subunit may also participate in the TNF signalling pathway since it interacts with the tumor necrosis factor type 1 receptor.
RFC4	It's a protein associated with BRCA1 which may serve as a sensor of abnormal DNA structures and/or as a regulator of the postreplication repair process [Wang et al. 2000]
EGFR*	Binding of the encoding protein to a ligand induces receptor dimerization and tyrosine autophosphorylation and leads to cell proliferation.
TSPAN1	Member of a family of proteins that mediate signal transduction events that play a role in the regulation of cell development, activation, growth and motility. This encoded protein is thought to be involved in growth-related cellular processes. This gene is associated with tumorigenesis and osteosarcoma.
EIF4G1	Using comparative genomic hybridization, Brass et al. (1997) identified amplification at 3q26.1-q26.3 in 30% of squamous cell carcinomas of the lung. In this way they identified 17 antigens that induced an immune response in a patient with squamous cell carcinoma.<br>Silvera et al. (2009) found that EIF4G1 was overexpressed in a significant number of inflammatory breast cancers. Silencing of EIF4G1 in SUM149 breast cancer cells via short hairpin RNA reduced EIF4G1-dependent translation of p120 mRNA, resulting in reduced cell surface E-cadherin expression and reduced tumorigenic potential of SUM149 cells.
PIK3CA*	PI 3-Kinases are responsible for coordinating a diverse range of cell functions including proliferation, cell survival, degranulation, vesicular trafficking and cell

	migration

	**DELETIONS**
**Gene**	**Description**
CDKN2A*	Capable of inducing cell cycle arrest in G1 and G2 phases. Acts as a tumor suppressor.
MLLT3*	This gene fuses with the ALL1 gene in leukemia-associated translocations
ENO1	Encoded protein binds to the myc promoter and acts as a transcriptional repressor. May be a tumor suppressor.
PLAA	Encoded protein is potentially important in regulating the inflammatory response through its activation of phospholipase A2.
ELAVL2	It has significant similarity to the product of the Drosophila elav gene, the absence of which causes multiple structural defects and hypotrophy of the fly's central nervous system. It is one of the tumor antigens that underlie paraneoplastic neurologic disorders (PND), which arise when an immune response to systemic tumors expressing neuronal proteins ('onconeural antigens') develops into an autoimmune neuronal degeneration.
FAF1	Potentiates but cannot initiate FAS-induced apoptosis : Interaction of Fas ligand (TNFSF6) with the FAS antigen (TNFRSF6) mediates apoptosis in a number of organ systems, and the protein encoded by FAF1 binds to FAS antigen and can initiate apoptosis or enhance apoptosis initiated through FAS antigen.
MAP3K4	MEKK4 is a major mediator of environmental stresses that activate the CSBP2 MAPK pathway, and a minor mediator of the JNK pathway. Component of a protein kinase signal transduction cascade. Activates the CSBP2, P38 and JNK MAPK pathways but not the ERK pathway. Specifically phosphorylates and activates MAP2K4 and MAP2K6
HRAS*	This gene belongs to the Ras oncogene family, whose members are related to the transforming genes of mammalian sarcoma retroviruses. The products encoded by these genes function in signal transduction pathways.
CARS*	This gene encodes a class 1 aminoacyl-tRNA synthetase, cysteinyl-tRNA synthetase. This gene is one of several located near the imprinted gene domain on chromosome 11p15.5, an important tumor-suppressor gene region.
NF1*	This gene product appears to function as a negative regulator of the ras signal transduction pathway. Mutations in this gene have been linked to neurofibromatosis type 1, juvenile myelomonocytic leukemia and Watson syndrome.
PCDH9	This is a cadherin-related neuronal receptor that localizes to synaptic junctions and is putatively involved in specific neuronal connections and signal transduction.
NUAK1	The authors proposed that ARK5 is a tumor cell survival factor that is activated by AKT and acts as an ATM kinase under conditions of nutrient starvation. [suzuki et al 2009] involved in tolerance to glucose starvation. Phosphorylates ATM. Suppresses Fas-induced apoptosis by phosphorylation of CASP6, thus suppressing the activation of the caspase and the subsequent cleavage of CFLAR
RFC4	BRCA1 is part of a large multisubunit protein complex of tumor suppressors, DNA damage sensors, and signal transducers. This complex was called BASC, and among them, there is the RFC4. Wang et al. (2000) suggested that BASC complex may serve as a sensor of abnormal DNA structures and/or as a regulator of the postreplication repair process.
PAOX	Plays an important role in the regulation of polyamine intracellular concentration and has the potential to act as a determinant of cellular sensitivity to the antitumor polyamine analogs

ELAVL4	Protects CDKN1A mRNA from decay by binding to its 3'-UTR.Manohar et al. (2002) suggested that HUD may contribute to the malignant phenotype of neuroblastoma cells by stabilizing MYCN mRNA, thereby enhancing steady-state levels of expression of this oncogene.
CAMTA1	Transcriptional activator. May act as a tumor suppressor (http://www.genecards.org/cgi-bin/carddisp.pl?gene=CAMTA1&search=CAMTA1)
