# Supplementary material for: Oncodrive-CIS: A Method to Reveal Likely Driver Genes Based on the Impact of Their Copy Number Changes on Expression
Source: PLoS One. 2013 Feb 8;8(2):e55489. doi: 10.1371/journal.pone.0055489 (PMC3568145; doi:10.1371/journal.pone.0055489)
Supplement: Table S3 — Same as Table S2 but for the Oncodrive-CIS results obtained in the ovarian serous carcinoma data set. (PDF) [file pone.0055489.s012.pdf]

Table S3.

Same as Table S2 but for the Oncodrive-CIS results obtained in the ovarian serous carcinoma data set.

	**AMPLIFICATIONS**
**Gene**	**Description**
ATAD2	May be a transcriptional coactivator of the nuclear receptor ESR1 required to induce the expression of a subset of estradiol target genes, such as CCND1, MYC and E2F1. May play a role in the recruitment or occupancy of CREBBP at some ESR1 target gene promoters. May be required for histone hyperacetylation. Involved in the estrogen-induced cell proliferation and cell cycle progression of breast cancer cells. [PubMed: 17998543]<br>Up-regulated in breast, uterus, colon, ovary, and stomach tumors. Induced in breast cancer cells overexpressing NCOA3 or treated with estrogen. Down-regulated in 5-fluorouracil-resistant derivatives of the colon cancer cell line HCT116 [[PubMed: 15334068][PubMed: 16709241] [PubMed: 17998543]]
CKAP2	Involved in regulating aneuploidy, cell cycling, and cell death in a p53/TP53-dependent manner. Jeon et al. (2006): Knockdown of CKAP2 reduced RB1 phosphorylation and increased p27 expression, and consequently reduced cell proliferation, whereas constitutive CKAP2 expression increased RB phosphorylation and enhanced proliferation.
BOP1	Strezoska et al. (2000): expression of an N-terminally truncated Bop1 protein (Bop1-delta) led to cell growth arrest in the G1 phase. Using small interfering RNA, Killian et al. (2004) found that downregulation of BOP1 and other components of the pescadillo complex involved in ribosome biogenesis altered chromosome segregation and led to abnormal mitoses in a human colon carcinoma cell line. Killian et al. (2006) concluded that deregulation of BOP1 can contribute to colorectal tumorigenesis.
CCNE1*	This cyclin forms a complex with and functions as a regulatory subunit of CDK2, whose activity is required for cell cycle G1/S transition.
GNL3	The protein encoded by this gene may interact with p53; may be required to maintain the proliferative capacity of stem cells and may play an important role in tumorigenesis .<br>Tsai and McKay (2002) suggested its role in maintaining stem cell self-renewal. Depletion or overexpression of nucleostemin reduces cell proliferation in CNS stem cells and transformed cells.<br>Increased levels in lung tissue in cancer patients [PubMed: 16012751].<br>May be required to maintain the proliferative capacity of stem cells and may play an important role in tumorigenesis.[PubMed: 16012751]]PubMed: 12464630]
RFC4	See Supp Table 2
RAD21	Cleavable component of the cohesin complex, involved in chromosome cohesion during cell cycle, in DNA repair, and in apoptosis.<br>RAD21 expression confers poor prognosis and resistance to chemotherapy in high grade luminal, basal and HER2 breast cancers [PMID: 21255398]<br>Some data suggest that KIAA0196 and possibly RAD21 are putative target genes for the common amplification of 8q23-24 in prostate cancer.[PMID:14603436]
PAK2	Pak kinases are thought to play critical roles in cell migration and invasion. Activation of PAK2 by RAC or CDC42 stimulates cell survival, whereas caspase-activated PAK2p34 induces a cell death response.<br>Pak1 and Pak2 mediate tumor cell invasion [PMID:18411304]<br><br>Highly expressed PAK2 mediates chemotherapeutic resistance in human breast invasive ductal carcinoma by negatively regulating caspase-7 activity [PMID:21555521].<br>Other data suggest that Pak1, p-Pak1 and p-Pak2 play important roles in ovarian carcinogenesis.[PMID:19876919]

	**DELETIONS**
**Gene**	**Description**
PPP2CB	It is generally considered to be a cancer suppressor as its inhibition can induce phosphorylation and activation of substrate kinases that mainly accelerate growth [PMID:21958460]
GLTSCR2	Okahara et al. (2004) confirmed the interaction between PTEN and GLTSCR2, the latter promoting the phosphorylation and stability of the former. Sasaki et al. (2011) concluded that this gene is a potent regulator of the MDM2-p53 pathway.
PTEN*	Tumor suppressor modulating cell cycle progression and cell survival.
CDKN2A*	See Supp Table 2
INTS9	Plays a role in small nuclear RNA processing. Dominski et al. (2005) showed the interaction between RC74 and RC68 in HeLa cells and mouse myeloma cells.
CLUAP1	May play a role in the control of cell survival. CLUAP1 expression is frequently misregulated in colon cancer.[Takahashi M et al 2004] and other malignancies including ovarian, colon, and lung cancers [Int J Oncol 2007]
ARHGAP10	Stabilizes PAK-2p34 thereby increasing stimulation of cell death [Koeppel et al. (2004)] Among invasive breast cancer GWAS, the most significant single-nucleotide polymorphism was located in ARHGAP10 [Pubmed: 20332263]
MTMR9	The encoded protein contains a double-helical motif similar to the SET interaction domain, which is thought to have a role in the control of cell proliferation. This gene demonstrated prognostic significance for oesophageal adenocarcinoma [PMID:21478220]
NF1*	See Supp Table 2
NCOA4*	Enhances the androgen receptor transcriptional activity in prostate cancer cells.
PLXNB2	Interaction with multiple upstream activators can finely tune the invasive growth process both in physiological conditions and in tumor growth and metastatization. [Oncogene. 2004 Jul 1;23(30):5131-7.]
POLDIP3	Richardson et al. (2004): RNAi-mediated reduction of S6K1 in HEK293 cells resulted in decreased POLDIP3 phosphorylation, and reduction of POLDIP3 by RNAi in human osteosarcoma cells resulted in decreased cell size, suggesting that POLDIP3 is a target of S6K1 in cell growth control.
DAD1	DAD1, the defender against apoptotic cell death, was initially identified as a negative regulator of programmed cell death in the temperature sensitive tsBN7 cell line. The DAD1 protein disappeared in temperature-sensitive cells following a shift to the nonpermissive temperature, suggesting that loss of the DAD1 protein triggered apoptosis.
